# Quaternary Alkylammonium Conjugates of Steroids: Synthesis, Molecular Structure, and Biological Studies

**DOI:** 10.3390/molecules201119735

**Published:** 2015-11-23

**Authors:** Bogumił Brycki, Hanna Koenig, Tomasz Pospieszny

**Affiliations:** Laboratory of Microbiocides Chemistry, Faculty of Chemistry, Adam Mickiewicz University, Umultowska 89b, 61-614 Poznań, Poland; koenig@amu.edu.pl (H.K.); tposp@amu.edu.pl (T.P.)

**Keywords:** squalamine, bile acids, sterols, quaternary alkylammonium salt, conjugates, prediction of activity spectra for substances (PASS), PM5 calculations

## Abstract

The methods of synthesis as well as physical, spectroscopic (^1^H-NMR, ^13^C-NMR, and FT-IR, ESI-MS), and biological properties of quaternary and dimeric quaternary alkylammonium conjugates of steroids are presented. The results were contrasted with theoretical calculations (PM5 methods) and potential pharmacological properties (PASS). Alkylammonium sterols exhibit a broad spectrum of antimicrobial activity comparable to squalamine.

## 1. Introduction

Steroids are an enormous group of very important natural products. The most significant compounds of this group are sterols (cholesterol, ergosterol, stigmasterol), bile acids (lithocholic, deoxycholic, cholic), and hormones (testosterone, estrogens, progesterone) [1,2,3,4,5]. Sterols are crucial constituents of the cell membrane of eukaryotes. Bile acids are amphipathic molecules with large, curved and rigid skeletons; chirality as well as the specific orientation of their chemically different polar hydroxy groups play an important role in metabolic processes. In turn, hormones determine the characteristics of sex and regulate pregnancy in animals, while plant hormones (brassinosteroids) cause elongation of stems and stimulate cell division (e.g., brassinolide) [6].

Another class of compounds that are involved in many biological processes are polyamines (spermidine, spermine, putrescine, cadaverine) [7,8,9,10]. Some of these are very important plant hormones and coenzymes.

**Figure 1:**
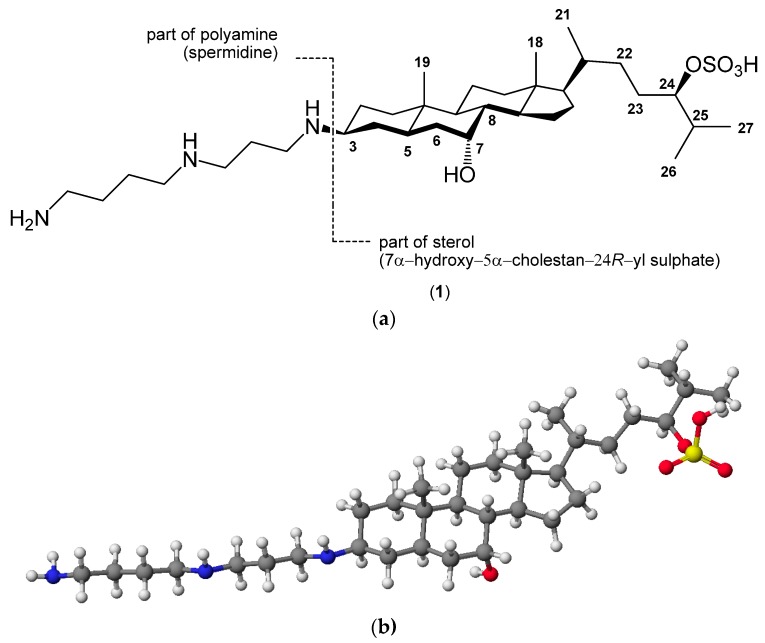
(**a**) The stereochemistry and numbering of squalamine and (**b**) a molecular model calculated by the PM5 method.

The connection of steroids and biogenic amines give the new conjugates unusual biological properties. The best-known compound of this type is squalamine (3β-spermidine-7α-hydroxy-5α-cholestan-24*R*-yl sulphate) (1) (Figure 1). The steroid–polyamine conjugate was isolated from the liver tissues of the dogfish shark (*Squalus acanthias*) [11,12,13,14]. This aminosterol is a novel broad-spectrum antibiotic and exhibits a biocidal activity against Gram-positive and Gram-negative bacteria, fungi, protozoa, and viruses [15,16,17,18,19,20,21,22,23]. The antimicrobial activity of the squalamine has inspired work to design and synthesize new derivatives of steroidal–polyamine conjugates [24,25,26,27,28,29,30,31,32].

## 2. Quaternary Alkylammonium Conjugates of Steroids

The basic criteria for the synthesis of biologically active conjugates of steroids and polyamines have been given by Salunke *et al.* [11]. Firstly, the structure must have a rigid extensive hydrophobic part and a flexible hydrophilic chain with a polar head group attached to a hydrophobic part. Secondly, the sulfate groups can be removed or replaced by a hydroxyl or carboxylate group. In turn, the structure of the polyamine is not important, and parts of steroids can be modified in various ways.

**Scheme 1:**
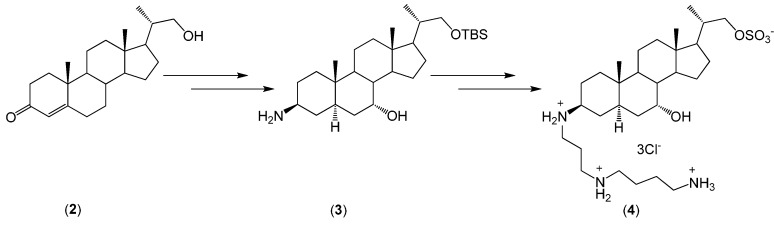
Synthesis of analogue of squalamine (**4**) from bisnoralcohol (**2**).

On this basis, Kim *et al.* described the synthesis of a squalamine analogue from bisnoralcohol (**2**) (Scheme 1) [16]. The structure of the product was confirmed by ^1^H-NMR, ^13^C-NMR, DEPT, COSY, HETCOR, and FT-IR, as well as low- and high-resolution mass spectra. Additionally, the biological activity of (**4**) has been determined. The squalamine analogue shows biocidal activity against *M. luteus* 9341, *S. aureus* 6538P, *K. pneumoniae* 10031, *S. equi* 6580C, and *B. subtilis* 6633. However, *E. coli* 25922, *P. aeruginosa* 27853, *P. mirabilis* 25933, *S. marcescens* 27117, and *S. typhimurium* 14028 are not sensitive to (**4**). In general, the antimicrobial activity of compound (**4**) is weaker in comparison to the antibacterial activity of squalamine.

**Figure 2:**
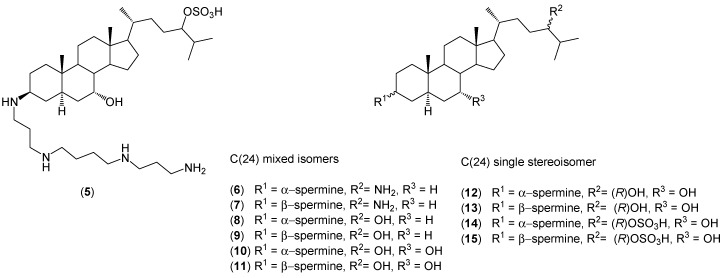
The structure of MSI-1436 (**5**) and its synthesized analogs (**6**–**15**).

Other analogs (**6**–**15**) of MSI-1436 (**5**) have been synthesized from stigmasterol by Shu *et al.* (Figure 2) [33]. The multistep reactions gave final products with very good yields. All analogs exhibit a broad spectrum of antimicrobial activity, which strongly depend on the stereochemistry of C(7) and C(3). By contrast, the stereochemistry at the C(24) has a negligible effect on the antibacterial activity.

**Figure 3:**
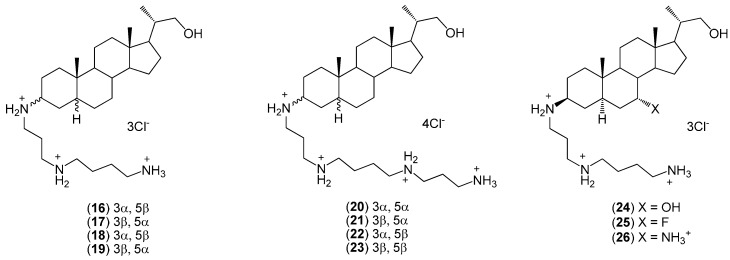
The structures of steroid–polyamine conjugates (**16**–**26**).

Similarly, Kim and co-workers focused on the effect of stereochemistry at the C(3) and C(5) atoms of steroids’ skeleton, as well as the types of polyamine attached to C(3) on activity against various human pathogens (Figure 3) [34,35,36,37]. The results showed that the stereochemistry of the C(3) and C(5) carbon atoms has a significant influence on the antimicrobial activity. For example, 3α-spermidine-23,24-bisnor-5α-cholane (**16**) was found to be more active than other spermidine analogues (**16**–**19**). However 3β-spermine-23,24-bisnor-5β-cholane (**23**) exhibits the highest biological activity among all the compounds (**16**–**23**). The conjugate (**17**), which is similar to (**24**–**26**) with the exception of the functional group at position C(7), has comparable antimicrobial activity to (**25**). Both compounds were much more active than the compounds (**24**) and (**26**). All synthesized conjugates (**16**–**26**) exhibited very good activity against Gram-positive bacteria.

**Scheme 2:**
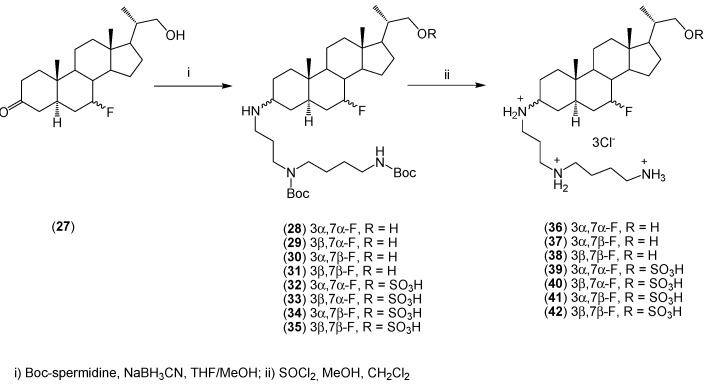
Synthesis of 7-fluoro-3-aminosterols (**36**–**42**).

The synthesis of a series of 7-fluoro-3-aminosteroids (**36**–**42**) is shown in Scheme 2 [37]. These compounds demonstrate a high antimicrobial activity, especially against *Staphylococcus aureus*, *Pseudomonas aeruginosa*, *Streptococcus pyogenes*, and *Escherichia coli* (Table 1).

**Table 1:** Minimum inhibitory concentrations (MIC, μg/mL) of 7-fluoro-3-aminosterols [37].

Microorganisms	Conjugate/MIC (μg/mL)							
	25	36	37	38	39	40	41	42
*S. pyogenes* 308A	6.3	25.0	12.5	12.5	12.5	50.0	25.0	50.0
*S. pyogenes* 77A	6.3	12.5	6.3	12.5	12.5	50.0	12.5	50.0
*S. ureus* 503	6.3	12.5	6.3	6.3	12.5	25.0	6.3	50.0
*E. coli* DC2	6.3	50.0	12.5	12.5	25.0	50.0	25.0	25.0
*P. aeruginosa* 9027	6.3	50.0	12.5	12.5	25.0	12.5	50.0	50.0
*P. aeruginosa* 1771M	3.1	100.0	25.0	6.3	50.0	25.0	50.0	50.0
*S. typhimurium*	100.0	100.0	50.0	100.0	100.0	100.0	50.0	50.0
*E. cloacae* 1321E	100.0	100.0	100.0	100.0	100.0	100.0	50.0	50.0

Great efforts have also been made to synthesize squalamine. Okumura *et al.* synthesized squalamine from a derivative of desmosterol via 12 steps with 7.4% of the total yield [38]. Moriarty and co-workers synthesized (**1**) from 3β-acetoxy-5-cholenic acid by 17 steps [39,40]. Jones *et al.* described a practical synthesis of squalamine from stigmasterol in 15 steps [41,42]. An excellent review of methods for the synthesis of spermine and spermidine analogues of squalamine is made by Brunel and Letourneux [43]. They reviewed the synthesis of squalamine from cholestane and dinorcholenic acid and described its biological activity and clinical perspectives.

**Figure 4:**
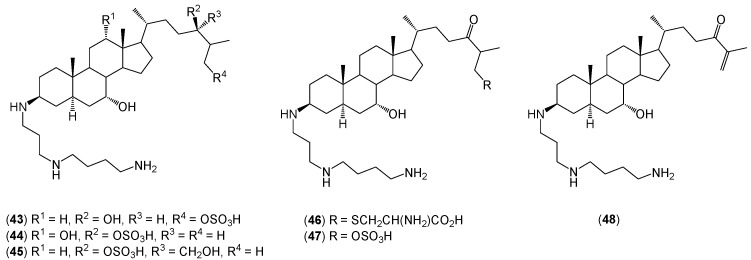
The structures of aminosterols (**43**–**48**) isolated from the dogfish shark.

In turn, Rao and co-workers isolated six other aminosterols (**43**–**48**) from the liver of the dogfish shark (Figure 4) [15]. The authors presented a very accurate spectral analysis based on 2D NMR (COSY, HETCOR, HMBC) as well as low- and high-resolution mass spectra (FAB, ESI, MALDI). The antimicrobial activity of aminosterols (**43**–**48**) and squalamine (**1**) is summarized in Table 2.

**Table 2:** Minimum inhibitory concentrations (MIC) of 3β-aminosterols [15].

Microorganisms	Conjugates/MIC (μg/mL)						
	1	43	44	45	46	47	48
*S. aureus* (29213)	1	4–8	8–16	2	8–16	8	2
*E. coli* (25922)	4	128	16	8	256	128	16
*P. aeruginosa* (27853)	16	32	16	16	256	128	16
*C. albicans* (90028)	16	16	32	32	128	32	2

**Scheme 3:**
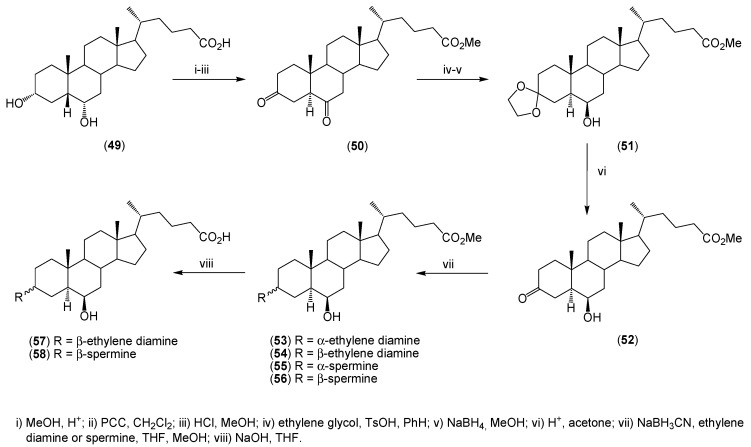
Synthesis of analogues of squalamine (**53**–**58**) from hyodeoxycholic acid (**49**).

Synthesis of 6β-hydroxy-3-α-(or β-)aminosterols (**53**–**58**) from hyodeoxycholic acid (**49**) has been presented by Jones *et al.* (Scheme 3) [44]. The modification of hyodeoxycholic acid was carried out by the esterification of the carboxyl group and oxidation of both hydroxyl groups to ketones, followed by a conversion of the A/B ring system from *cis* to *trans* by acid-catalyzed isomerization. Then various polyamines were added and the corresponding stereoconjugates were obtained.

The synthesized aminosterol conjugates (**53**–**58**) exhibit a broad spectrum of antimicrobial activity, similar to other aminosterols (Table 3).

**Table 3:** Minimum inhibitory concentrations (MIC, μg/mL) of 3α (or 3β)-aminosterols [44].

Microorganisms	Conjugates/MIC (μg/mL)						
	1	53	54	55	56	57	58
*S. aureus*	0.5–1	16	1	2–4	2	>256	16
*E. coli*	2–4	32–64	8–16	32	32	>256	16
*P. aeruginosa*	16	128	64	128	32	128	8
*C. albicans*	8	8	2–4	4	2	>256	4

The presented data show that the β-analogs (**54**, **56**) are slightly more active against microorganisms than the α-analogs (**53**, **55**). Moreover, the biocidal efficacy against *S. aureus* is higher for methyl esters (**54**, **56**) in comparison to free acids (**57**, **58**). The chain length of the polyamine has no significant effect on biocidal activity. However, for acid derivatives, a conjugate with spermine chain (**58**) was much more active than a conjugate with an ethylene diamine chain (**57**).

**Scheme 4:**
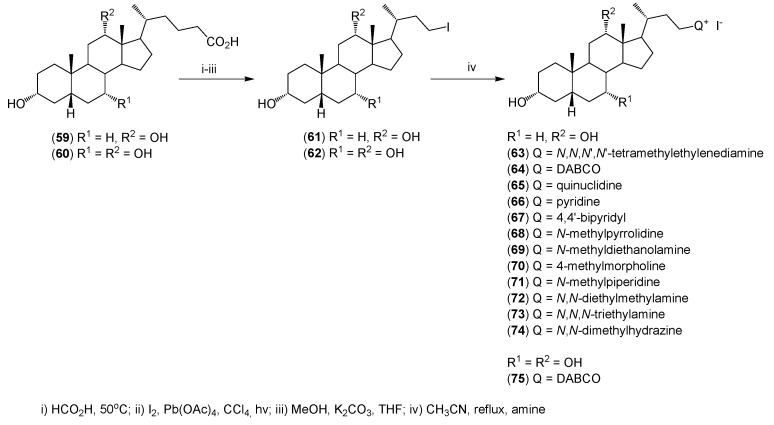
Synthesis of cationic bile salts from iodo derivatives of bile acids.

Maitra *et al.* used their own method to modify the side chain of bile acids [45,46]. The synthesis of quaternary alkylammonium conjugates of bile acids (**63**–**75**) is shown in Scheme 4.

Bile acids (**59**, **60**) were transformed to the 24-nor-23-iodo (**61**, **62**) derivatives by a Hunsdiecker reaction followed by a reaction with secondary or tertiary amines, respectively. All conjugates (**63**–**75**) were obtained with good yields 65%–75% and were characterized by ^1^H-NMR, ^13^C-NMR, and FT-IR, as well as mass spectrometry. These quaternary ammonium conjugates were found to be good gelators. Some of the quaternary ammonium bile salts gelled water and many of them gelled aqueous salt solutions even in the presence of organic solvents such as alcohol (methanol, ethanol) as well as DMF or DMSO. These gels form fibrous networks [46].

**Scheme 5:**
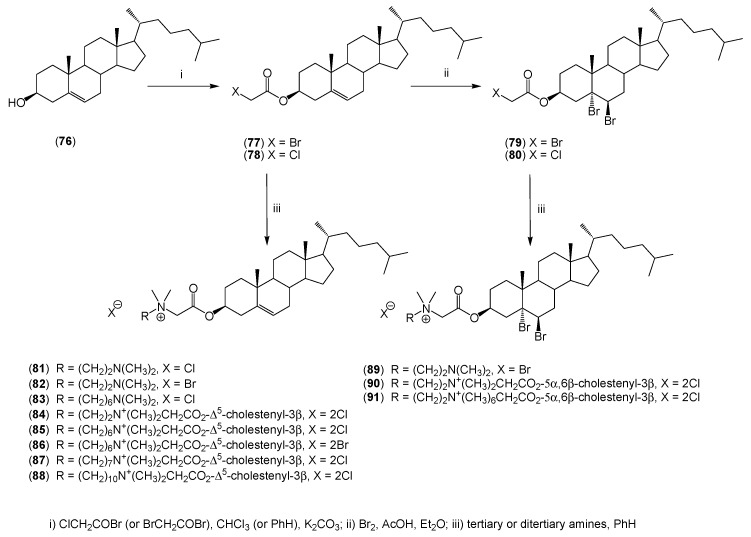
Synthesis of monoquaternary (**81**–**83**, **89**) and symmetrical bisquaternary salt (**84**–**88**, **90**, **91**) derivatives of cholesterol.

Lopushanskii and Udovitskaya described the method to prepare cholesteryl 3β-bromoacetate and 3β-chloroacetate, which were used in the synthesis of quaternary ammonium derivatives of cholesterol and its 5α,6β-dibromo derivatives (**81**–**91**) (Scheme 5) [47].

**Figure 5:**
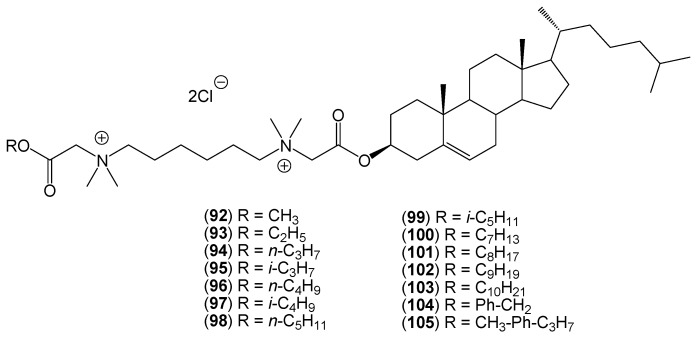
The structures of unsymmetrical bisquaternary salt (**92**–**105**) derivatives of cholesterol.

In addition to monoquaternary salts (**81**–**83**) and (**89**), as well as symmetrical bisquaternary salts (**84**–**88**) and (**90**, **91**), the authors obtained and described unsymmetrical bisquaternary salts (**92**–**105**) (Figure 5). The unsymmetrical bisquaternary ammonium salts (**92**–**101**) demonstrate a bacteriostatic activity that depends on the alkyl chain length.

**Figure 6:**
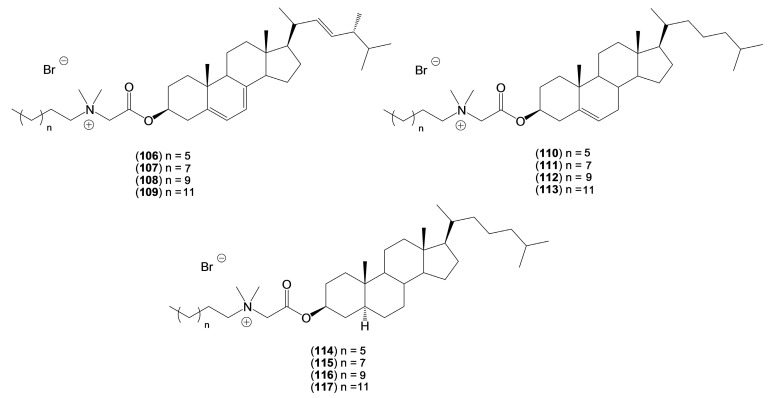
The quaternary alkylammonium conjugates of sterols (**106**–**117**).

Brycki and co-workers obtained the series of quaternary alkylammonium conjugates of ergosterol, cholesterol, and cholestanol [48]. The conjugates were synthesized by two-step reactions. In the first step ergosterol, cholesterol, and cholestanol were reacted with bromoacetic acid bromide with TEBA and calcium hydride (or sodium hydride) in anhydrous toluene to give 3β-bromoacetates of sterols [49]. In the second step, 3β-bromoacetates have been treated with tertiary alkylamines (CH_3_–(CH_2_)_n_–N(CH_3_)_2_, *n* = 7, 9, 11, 13) under S_N_2 reaction conditions to give conjugates of ergosterol (**106**–**109**), cholesterol (**110**–**113**), and cholestanol (**114**–**117**) (Figure 6).

**Figure 7:**
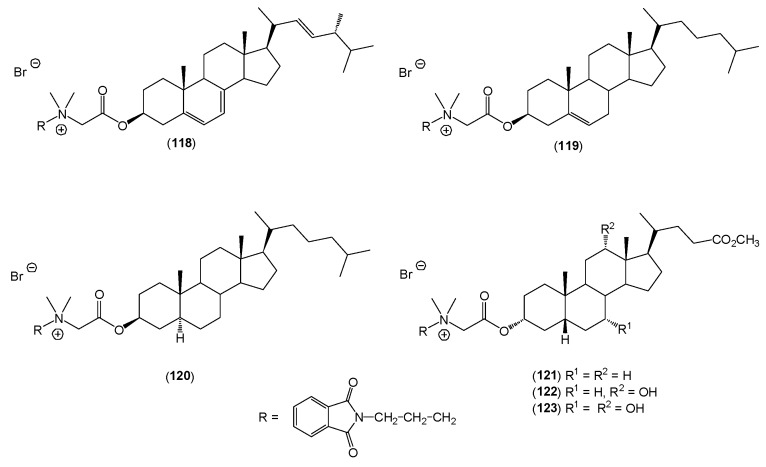
*N*,*N*-dimethyl-3-phthalimidopropylammonium conjugates of sterols (**118**–**120)** and bile acids (**121**–**123**).

The authors also obtained a series of *N*,*N*-dimethyl-3-phthalimidopropylammonium conjugates of sterols (ergosterol, cholesterol, cholestanol) (**118**–**120**) and bile acids (lithocholic, deoxycholic, cholic) (**121**–**123**) (Figure 7) [50]. The synthesis and physicochemical properties of quaternary *N*,*N*-dimethyl-3-phthalimidopropylammonium conjugates of ergosteryl 3β-bromoacetate, cholesteryl 3β-bromoacetate, and dihydrocholesteryl 3β-bromoacetate, as well as methyl litocholate 3α-bromoacetate, methyl deoxycholate 3α-bromoacetate, and methyl cholate 3α-bromoacetate with *N*,*N*-dimethyl-3-phthalimidopropylamine in acetonitrile were investigated and described.

**Figure 8:**
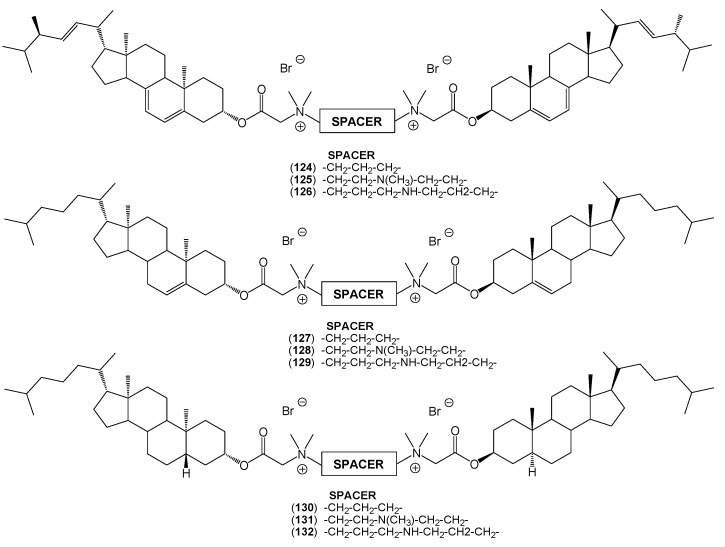
The symmetrical bisquaternary alkylammonium conjugates of sterols (**124**–**132**).

The symmetrical dimeric quaternary alkylammonium conjugates of sterols (**124**–**132)** prepared by two-step reactions of ergosterol, cholesterol, or cholestanol with bromoacetic acid bromide, followed by bimolecular nucleophilic substitution with *N*,*N*,*N'*,*N'*-tetramethyl-1,3-propanediamine, *N*,*N*,*N'*,*N''*,*N''*-pentamethyldiethylenetriamine, and 3,3′-iminobis-(*N*,*N*-dimethylpropylamine) have been also described by Brycki *et al.* (Figure 8) [51]. The final reactions were carried out in acetonitrile to favor bimolecular nucleophilic substitution and optimize the reaction yields.

**Figure 9:**
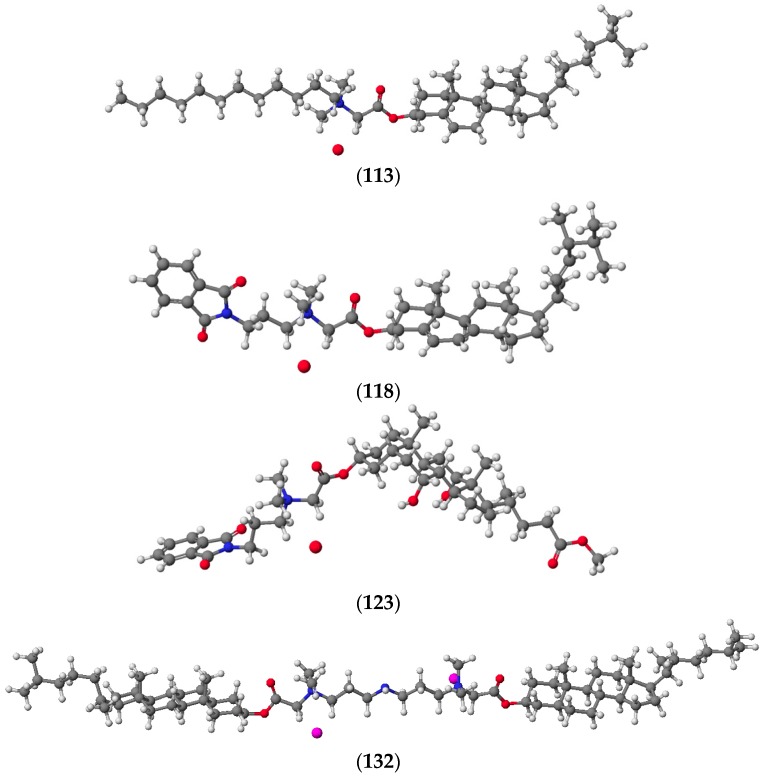
The representative quaternary alkylammonium conjugates of sterols calculated by the PM5 method.

All structures of the conjugates were confirmed by spectral (^1^H-NMR, ^13^C-NMR, and FT-IR) analysis and mass spectrometry as well as theoretical semiempirical methods (PM5). PM5 semiempirical calculations were performed using the WinMopac 2003 program [52,53,54]. In all cases, the heat of formation (HOF) was consistent with the expected values. The lowest values of HOF for sterols were observed for conjugates of cholestanol (**114**–**117**, **120**, **130**–**132**) where there were no double bonds to stabilize the molecule and hinder its reactivity. This was in contrast to conjugates of ergosterol (**106**–**109**, **118**, **124**–**126**) and cholesterol (**110**–**113**, **114**, **127**–**129**), where the double bonds increase the reactivity of the molecule, thereby increasing values of HOF (Figure 9). In turn, the HOF of conjugates of methyl esters of bile acids (**121**–**123**) can be explained in a similar manner. For these compounds the number of hydroxyl groups in the steroid skeleton lowers the value of HOF.

The potential pharmacological activities of the synthesized compounds have been studied using a computer-aided drug discovery approach with the *in silico* Prediction of Activity Spectra for Substances (PASSs) program. It is based on a robust analysis of the structure–activity relationships in a heterogeneous training set currently including about 60,000 biologically active compounds from different chemical series with about 4500 types of biological activities. Since only the structural formula of the chemical compound is necessary to obtain a PASS prediction, this approach can be used at the earliest stages of investigation. There are many examples of the successful use of the PASS approach leading to new pharmacological agents [55,56,57,58,59]. The PASS software is useful for the study of the biological activity of secondary metabolites. The types of activities that were predicted for a potential compound with the highest probability (focal activities) have been selected. If predicted activity (PA) > 70, the substance is very likely to exhibit experimental activity and the chance of the substance being the analogue of a known pharmaceutical agent is also high. If 50 < PA < 70, the substance is unlikely to exhibit the activity in experiment, the probability is less, and the substance is unlike any known pharmaceutical agent. A research group led by Brycki selected the types of activity that were predicted for a potential compound with the highest probability (Table 4).

**Table 4:** Probability “to be Active” (PA) values for predicted biological activity of compounds (**106**–**132**).

Focal Predicted Activity (PA > 80)	Conjugates																	
	106–109	110–113	114–117	118	119	120	121	122	123	124	125	126	127	128	129	130	131	132
Cholesterol antagonist	88	90	87	–	–	–	–	–	–	81	85	–	87	89	82	82	86	–
Antihypercholesterolemic	91	87	–	–	–	–	–	–	–	88	83	86	85	80	83	–	94	–
Glyceryl-ether monooxygenase inhibitor	89	92	95	87	91	93	93	94	95	89	89	88	92	92	91	95	95	94
Acylcarnitine hydrolase inhibitor	–	87	97	–	–	81	83	91	94	–	–	–	85	80	–	96	–	93
Alcohol *O*-acetyltransferase inhibitor	91	–	–	–	–	–	–	–	–	91	90	90	–	–	–	–	–	–
Oxidoreductase inhibitor	81	–	–	–	–	–	–	–	–	87	86	85	–	–	–	–	–	–
Prostaglandin-E2 9-reductase inhibitor	–	86	–	–	–	–	–	–	–	–	–	–	–	–	–	–	–	–
Alkylacetylglycerophosphatase inhibitor	–	–	92	–	–	84	82	90	86	–	–	–	–	–	–	90	87	83
Alkenylglycerophosphocholine hydrolase inhibitor	–	–	90	–	–	–	–	80	–	–	–	–	–	–	–	88	82	80

## 3. Conclusions

The design and preparation of new steroid conjugates allow us to develop the fields of supramolecular chemistry, material chemistry, and nanotechnology. In this paper we described the synthesis and physicochemical properties of quaternary alkylammonium conjugates of steroids. Most of the described compounds are characterized by high biological activity with a broad spectrum of antimicrobial and antifungal activity. Moreover, these compounds can actively participate in transport across biological membranes, which offers tremendous possibilities in biochemistry, pharmacology, and medicine. The spectroscopic data, semiempirical calculations, and potential pharmacological properties (PASS) obtained in this work significantly extend the library of new steroid conjugates.
